# Sinus Microbiota in Patients With Eosinophilic and Non-Eosinophilic Chronic Rhinosinusitis With Nasal Polyps

**DOI:** 10.3389/fcimb.2021.672355

**Published:** 2021-07-23

**Authors:** Tingting Feng, Ping Miao, Bin Liu, Yao Liu, Ximing Bao, Ji Xu, Nana Ren, Ying Li, Jiali Shi, Wanxin Cao, Jianchen Fang, Min Li, Qian Liu, Jiping Li

**Affiliations:** ^1^ Department of Otorhinolaryngology, Renji Hospital, School of Medicine, Shanghai Jiaotong University, Shanghai, China; ^2^ Department of Laboratory Medicine, Renji Hospital, School of Medicine, Shanghai Jiaotong University, Shanghai, China; ^3^ Department of Otorhinolaryngology, Peking University Third Hospital, Peking University, Beijing, China; ^4^ Department of Pathology, Renji Hospital, School of Medicine, Shanghai Jiaotong University, Shanghai, China

**Keywords:** microbiota, chronic rhinosinusitis with nasal polyps, eosinophil infiltration, 16S rRNA, *Staphylococcus aureus*

## Abstract

Chronic rhinosinusitis with nasal polyps (CRSwNP) is characterized by Th2-skewed inflammation and increased colonization by *Staphylococcus aureus*. CRSwNP can be distinguished as eosinophilic (ECRSwNP) and non-eosinophilic (NECRSwNP) by the infiltration of eosinophils. The local microbiota plays an important role in the persistent inflammation of CRSwNP. To evaluate the bacterial community composition on the distinct types of CRSwNP patients, we collected nasal swabs from 16 ECRSwNP patients, 18 NECRSwNP patients, and 39 healthy control subjects. The microbiome structure for all the samples were analyzed by high-throughput 16S rRNA gene sequencing. Concentration of *S. aureus* was determined using TaqMan quantitative polymerase chain reaction (qPCR) targeting the nuclease (*nuc*) gene. The result showed significant differences in the sinus microbiome among healthy control subjects and CRSwNP patients. Microbiota community diversity was significantly lower in NECRSwNP samples compared to that of healthy control subjects. Interestingly, the abundance of several pathogenic bacteria was diverse between ECRSwNP and NECRSwNP patients. Although *Staphylococcus* prevailed in all groups, the abundance of *Staphylococcus* was significantly higher in the healthy control group than the ECRSwNP group. More importantly, the abundance of *S. aureus* was much higher in NECRSwNP patients. This study highlights that microbiota composition may contribute to the different clinical types of CRSwNP, inspiring new therapeutic strategies to resolve this chronic inflammation process.

## Introduction

Chronic rhinosinusitis with nasal polyps (CRSwNP) is widespread around the world, with a prevalence of 2.1% in France (Klossek et al., 2005), 4.3% in Finland (Hedman et al., 1999), 4.2% in the United States (Settipane and Chafee, 1977), and 1.1% in China (Shi et al., 2015). CRSwNP is a heterogenous, multifactorial disease, characterized by persistent mucosal inflammation of the nasal cavity and paranasal sinuses. The symptoms of CRSwNP include anterior or posterior rhinorrhea, nasal congestion, hyposmia, and/or facial pressure or pain that lasts for greater than 12 weeks (Stevens et al., 2016). The tissue inflammatory response in CRSwNP patients is characterized by prominent eosinophilia with a Th2-skewed response. Despite medical treatment and surgical interventions, CRSwNP recurrences are frequent. The underlying mechanisms that contribute to the persistent inflammation of CRSwNP are not completely defined. It has been reported that the local microbiome contributes to the inflammation of CRSwNP (Ba et al., 2011; Chalermwatanachai et al., 2015). Under healthy circumstances, the airway microbiome has been recognized as part of the first line of defense, protecting mucosal surfaces from infection. However, dysbiosis and decreased complexity of the sinus microbiome, determining a shift in the balance between commensal and potentially pathogenic microorganisms, are well-recognized features in patients with CRS (Abreu et al., 2012; Ramakrishnan et al., 2015; Wagner Mackenzie et al., 2017; Chalermwatanachai et al., 2018). Several studies suggest that a higher bacterial load and/or a lower diversity in the airway microbiota may be associated with the inflammatory process (Feazel et al., 2012; Biswas et al., 2015; Chalermwatanachai et al., 2015; Ramakrishnan et al., 2015; Cope et al., 2017). Pathogenic bacteria including *Staphylococcus aureus*, *Moraxella catarrhalis*, *Streptococcus*, and so on are frequently recovered from patients with CRSwNP, indicating that bacteria colonization is involved in the inflammation of the disease (Van Zele et al., 2004; Vickery et al., 2019).

CRSwNP can be classified into two types: eosinophilic (ECRSwNP) and non-eosinophilic (NECRSwNP). Clinically, ECRSwNP patients often present with significant hyposmia and are usually responsive to corticosteroids, both at a systemic level and topically (Fujieda et al., 2019; Grayson et al., 2019). NECRSwNP patients normally present with nasal obstruction and purulent secretions and tend to have the greatest response to low-dose, long-term macrolide therapy (Oakley et al., 2018; McCormick et al., 2020). Research has confirmed that ECRSwNP usually exhibits severe eosinophilic infiltration, while NECRSwNP is characterized by neutrophil-predominant inflammation (Cao et al., 2009; Shi et al., 2013). Because of different inflammation responses, we hypothesized that the colonization of the local microbiome may be involved in the development of diseases.

Here, we investigated the sinus microbiota collected from the middle meatus of 34 CRSwNP (16 ECRSwNP and 18 NECRSwNP) and 39 healthy control subjects by 16S rRNA sequencing. Generally, we observed significant differences of microbiota composition between CRSwNP and healthy control subjects. The abundance of *Staphylococcus* is predominant in all groups. Although the microbiota composition overlapped between ECRSwNP and NECRSwNP, we observed significant differences for the abundance of several pathogenic bacteria. The abundance of *S. aureus* was the highest in the NECRSwNP group. Taken together, our data suggested that the microbiome composition is diverse in patients with different endotypes.

## Materials and Methods

### Patient Recruitment and Sample Collection

From July 2019 to January 2020, 73 subjects were recruited for this study in Renji Hospital, Shanghai. All subjects recruited in this study came from Southeast China, relative to Renji Hospital, Shanghai. Among them, 34 patients with CRSwNP underwent functional endoscopic sinus surgery (FESS), and 39 healthy control patients undertook an endoscopic procedure for indications other than CRS. The diagnosis of CRSwNP was based on the guidelines from the European Position Paper on Rhinosinusitis and Nasal Polyps (Fokkens et al., 2012). Patients with co-morbidities such as cystic fibrosis, ciliary immobile syndrome, aspirin exacerbated respiratory disease, immunodeficiency, allergic fungal sinusitis, and inverted papilloma were excluded. None of the patients took oral glucocorticoids, or antibiotics within 4 weeks before surgery. Demographic data and medical history were recorded for each patient. SinoNasal Outcomes Test 22 (SNOT-22) was used to measure patient-reported symptom severity. All patients underwent a high-resolution CT scan of the paranasal sinuses, and a standard Lund-Mackay scoring system was used to assess the overall extent of CRSwNP. Written informed consent was obtained from all the participants. The study design and materials were reviewed and approved by the Research Ethics Committee of Renji Hospital.

### Sample Collection

Samples from patients of the Department of Otorhinolaryngology at Renji Hospital were collected after receiving written informed consent for their inclusion. Swabs specimens for DNA extraction were endoscopically guided to the middle meatus region and rotated at least five times. Swabs were immediately submerged in 1 ml of sterile saline for DNA extraction. Sinus mucosal tissue samples were collected intraoperatively from the uncinate process under general anesthesia. Venous blood samples (2 mL) were collected and anti-coagulated with heparin. Swabs were immediately submerged in 1 ml of sterile saline for further use.

### Histological Analysis

Tissue samples were fixed in 4% buffered formalin, embedded in paraffin, cut into 4-um-thick sections, and stained with hematoxylin and eosin (H&E). Afterward, the stained sections were observed by two independent pathologists who were blind to the clinical data. The number of eosinophils was counted at high power (HP) magnification (*400), and three HP fields were randomly selected and analyzed. The tissue was reported as eosinophilic by the pathologists if eosinophils were found in more than 10% of inflammatory cells in the studied area (Cao et al., 2009; Hu et al., 2012; Cao et al., 2014; Mahdavinia et al., 2015; Ma et al., 2016).

### Cytokine Detection

Blood samples were subjected to centrifugation at 3,000 rpm for 20 min; the serum was collected and stored at -20°C for cytokines detection. The level of plasma cytokines (IFN-γ, TNF-α, IL-2, IL-4, IL-6, IL-10, and IL-17A) were detected using a Human Th1/Th2/Th17 Cytometric Bead Array kit (Sai Ji Bioscience, Inc. Jiangxi, China), according to the manufacturer’s instructions.

### DNA Extraction and 16S rRNA Gene Sequencing

Swabs were submerged in 1 mL of sterile saline and vortexed for 2 min. The 800 μl samples from each swab were centrifuged at 10000 rpm for 10 min. All samples were stored at −80°C until DNA extraction. DNA extraction was performed according to the protocol of the QIAamp DNA Mini Kit. The quality and concentration of the extracted DNA were measured using a NanoDrop spectrophotometer. The V1-V2 (Zheng et al., 2019) hypervariable region of the bacterial 16S rRNA gene was amplified by PCR using the following cycling parameters: 95°C for 3 min, 25 cycles at 95°C for 30 s, and 55°C for 30 s, 72°C for 30 s, and a final extension at 72°C for 5 min, with primers 27F (5′-AGAGTTTGATCCTGGCTCAG-3′) and 338R (5′-TGCTGCCTCCCGTAGGAGT-3′). The amplicons were subsequently purified by AMPure XP beads. The purified amplicons were pooled in equimolar and paired-end sequenced (2×250) on an Illumina MiSeq platform according to the standard protocols. All raw sequences obtained during this study were submitted to the NCBI Sequence Read Archive (accession number: PRJNA697755).

### Sequence Analysis

The overlapping regions between the paired-end reads were assembled using Fast Length Adjustment of SHort reads (FLASH) (Magoč and Salzberg, 2011). Low quality reads were filtered by fastq_quality_filter (-p 90 -q 25 -Q33) in FASTX Toolkit 0.0.14 and chimera reads were removed by USEARCH 64 bits v8.0.1517. The number of reads for each sample was normalized based on the smallest size of samples by random subtraction. OTUs were aligned by the UCLUST algorithm with a 97% identity and taxonomically classified using the SILVA 16S rRNA database v128. The alpha diversity was analyzed using the Quantitative Insights into Microbial Ecology (QIIME). Beta diversity was assessed with Principal Coordinates Analysis (PCoA) implemented by the QIIME pipeline and was calculated based on unweighted Unifrac distance matrices (Caporaso et al., 2010).

### TaqMan qPCR Assay

Concentrations of *S. aureus* DNA were determined using quantitative TaqMan real-time PCR targeting the single-copy gene. The nuclease (*nuc*) gene, which encodes an extracellular enzyme unique to *S. aureus*, is a widely used molecular target for the rapid detection of *S. aureus* and can also be used to determine *S. aureus* colonization status (Redel et al., 2013). Sequences of the primer were analyzed using the basic local alignment search tool (BLAST) (http://blast.ncbi.nlm.nih.gov/) against the GeneBank nucleotide database, to confirm that the primer set was specific to *S. aureus*. qPCR was performed in a 20 μl reaction volume containing 2x KAPA PROBE FAST qPCR Master Mix, 200 nM concentrations of primers *S. aureus nuc* F372 (TGTAGTTTCAAGTCTAAGTAGCTCAGCAA), *S. aureus nuc* R465 (TGCACTATATACTGTTGGATCTTCAGAA), TaqMan probe (TGCATCACAAACAGATAACGGCGTAAATAGAAG), and 4 μl of DNA template. The negative control contained sterile distilled water instead of template DNA. Amplification was performed on the 7500 Real-Time PCR system using the following cycling parameters: denaturation at 95°C for 3 min, 40 cycles at 95°C for 15 s, and 55°C for 30 s. Bacterial loads were determined using the standard curves. Samples and standard curves were run in triplicate. Samples giving cycle threshold (Ct) value s≤ 36 were considered positive for *S. aureus*.

### Statistical Analysis

The Kolmogorov–Smirnov test was used to evaluate the normality of data. Baseline characteristics are displayed as mean ± standard deviation unless otherwise indicated. Unpaired t-tests or Mann–Whitney U tests were used to calculate the statistical significance of differences between groups for continuous data. The Chi-square test with Fisher’s exact test were used to compare differences between groups for categorical data. Statistical significance of differences in α-diversity indices between groups was evaluated by one-way ANOVA with Tukey’s post-test. An ANOSIM test was used to visualize differences in principal coordinate analysis **(**PCoA) among groups. The Kruskal-Wallis test, followed by Dunn’s multiple comparisons test, were used to evaluate statistical differences in abundance of phylum, abundance of genus, and *S. aureus* burden among groups. All tests were two-tailed, and a *p*-value <0.05 was considered significant. Significances were expressed as *p < 0.05, **p < 0.01, ***p <0.001, and ****p < 0.0001. Statistical analysis and graphical outputs were performed using the Prism GraphPad version 8 software program (GraphPad Software, San Diego, California).

## Results

### Clinical Characteristics of the Patients

Seventy-three adults (39 healthy control subjects, 34 CRSwNP patients) were enrolled in the study. **Table 1** illustrates the participants’ demographic parameters. No significant differences were found among the three groups with respect to age and gender. Previous surgeries were significantly more prevalent in NECRSwNP patients, while asthma comorbidity was significantly more prevalent in ECRSwNP patients (**Table 1**).

**Table 1:** Demographic characteristics of healthy control and patient groups.

Parameter	HC	ECRSwNP	NECRSwNP	*P*-value
Total cases	39	16	18	
Male/female	18/21	11/5	7/11	*p=*0.181
Mean age (y)	39.16	48.31	28.50	*p*=0.792
Previous surgeries	0/39	2/16	6/18	***p*=0.001**
Asthma	0/39	2/16	0/18	***p*=0.046**

Statistically significant differences between groups were calculated using the Kruskal-Wallis test for continuous variables, Chi-square test with Fisher’s exact test were used for categorical data. The significant differences is marked with bold p (p<0.05). HC ,healthy control; ECRSwNP, eosinophilic chronic rhinosinusitis with nasal polyps; NECRSwNP, non-eosinophilic chronic rhinosinusitis with nasal polyps.

### Differences in Clinical Presentation Between ECRSwNP and NECRSwNP

The clinical characteristics of the study population are summarized in **Table 2**. We recorded two blood parameters (peripheral blood eosinophil and peripheral blood eosinophil percentage), SNOT-22 test, and patients’ computed tomography (CT) scan to examine whether there were differences between ECRSwNP and NECRSwNP. The Lund-Mackay scoring system, used as an opacification staging system, was developed for patients undergoing sinus surgery, representing the severe end of the disease spectrum. SNOT-22 was used as the reference questionnaire to assess symptoms, health-related quality-of-life, and treatment-response in patients with chronic rhinosinusitis. ECRSwNP patients showed a significantly higher degree of peripheral blood eosinophil (0.37 ± 0.24, 0.08 ± 0.08) and peripheral blood eosinophil percentage (5.94 ± 3.70, 1.35 ± 1.47) compared to NECRSwNP patients. Significant differences were observed between the ECRSwNP and NECRSwNP groups in total Lund-Mackay score (17.87 ± 10.24, 14.00 ± 7.59), maxillary sinuses (2.44 ± 1.37, 1.50 ± 1.20), anterior ethmoidal (3.25 ± 0.93, 2.00 ± 1.37), posterior ethmoidal (3.00 ± 1.03, 1.50 ± 1.34), frontal sinuses (2.50 ± 1.51, 1.33 ± 1.46), and ostiomeatal complex (3.50 ± 1.16, 1.22 ± 1.56). There was no significant difference in SNOT-22 score between the two groups.

**Table 2:** Clinical characteristics of patients with ECRSwNP and NECRSwNP.

Parameter	ECRSwNP	NECRSwNP	*p*-value
Peripheral blood eosinophil (10^9^/L)	0.37 ± 0.24	0.08 ± 0.08	***p=*0.000**
Peripheral blood eosinophil percentage (%)	5.94 ± 3.70	1.35 ± 1.47	***p=*0.000**
Total Lund-Mackay score	16.56 ± 5.97	8.56 ± 6.56	***p*=0.001**
Maxillary sinuses	2.44 ± 1.37	1.50 ± 1.20	***p*=0.042**
Anterior ethmoidal	3.25 ± 0.93	2.00 ± 1.37	***p*=0.009**
Posterior ethmoidal	3.00 ± 1.03	1.50 ± 1.34	***p*=0.002**
Sphenoidal sinuses	1.88 ± 1.59	1.00 ± 1.41	*p*=0.117
Frontal sinuses	2.50 ± 1.51	1.33 ± 1.46	***p*=0.036**
OMC	3.50 ± 1.16	1.22 ± 1.56	***p*=0.000**
Total SNOT-22 score	17.87 ± 10.24	14.00 ± 7.59	*p*=0.347
Nasal	11.19 ± 5.44	8.28 ± 4.50	*p*=0.126
Ear/facial discomfort	0.94 ± 1.44	1.22 ± 1.44	*p*=0.443
Sleep	5.25 ± 5.57	4.00 ± 3.29	*p*=0.932
Emotional	0.50 ± 1.10	0.50 ± 0.786	*p*=0.646

Data are the mean ± standard deviation (SD). ECRSwNP, eosinophilic chronic rhinosinusitis with nasal polyps; NECRSwNP, non-eosinophilic chronic rhinosinusitis with nasal polyps; SNOT-22, SinoNasal Outcomes Test-22; OMC, ostiomeatal complex. Statistically significant differences between groups were calculated using the Mann-Whitney test. The significant differences is marked with bold p (p<0.05).

CRSwNP is an inflammation state. To avoid the impact of inflammation on the local microbiota, cytokine profiling of the peripheral blood for all the patients was further analyzed. Levels of plasma cytokines (IFN-γ, TNF-α, IL-2, IL-4, IL-6, IL-10, and IL-17A) were detected and displayed a similar level between ECRSwNP and NECRSwNP groups (**Supplementary Table 1**).

### Microbiota Differences Among Healthy Control, ECRSwNP, and NECRSwNP Patients

**Figure 1:**
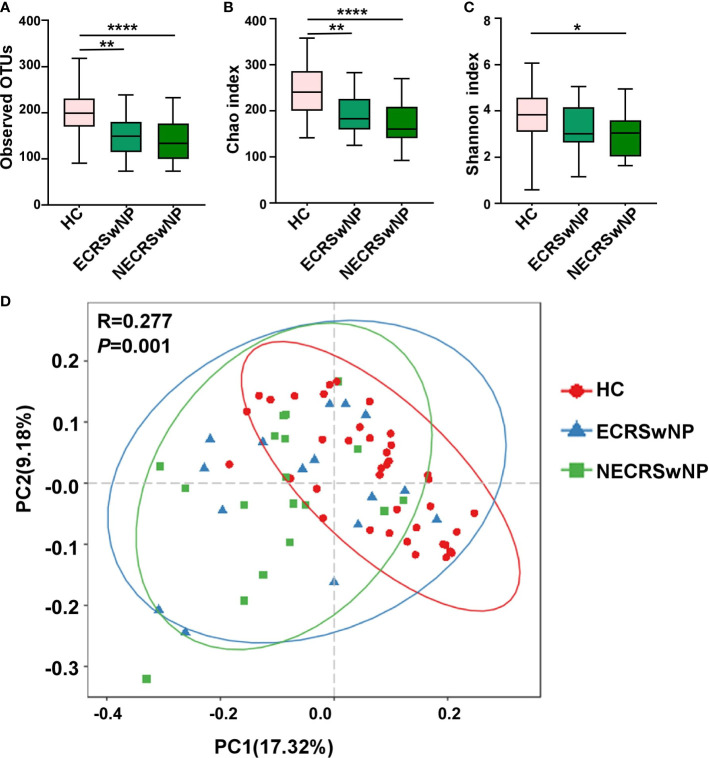
The alpha and beta diversity of the sinus microbiota among groups. **(A)** Number of observed OTUs; **(B)** Chao1 diversity index; **(C)** Shannon diversity index. n = 39 for healthy control, n = 16 for ECRSwNP, and n=18 for NECRSwNP. Whisker boxes are drawn from the first to third quartiles. Error bars show minima and maxima. ^*^ *p* < 0.05, ^**^ *p* < 0.01, ^****^ *p* < 0.0001, statistical evaluation was by one-way ANOVA with Tukey’s post-test. **(D)** PCoA plots in genus levels. Each point represents a sample and the distance between points indicates the similarity of those points. Statistical difference was calculated by the ANOSIM test. *p* = 0.01, R = 0.277. HC, healthy control; ECRSwNP, eosinophilic chronic rhinosinusitis with nasal polyps; NECRSwNP, non-eosinophilic chronic rhinosinusitis with nasal polyps.

To confirm that the number of reads for all samples were adequate for the following analysis, the rarefaction curves were computed first and the plateau was observed for all samples (**Supplementary Figure 1**). Alpha-diversity indices were calculated for all the subjects included in the study. Compared to the healthy control (HC) group, the sinonasal microbiota of the CRSwNP group showed significantly decreased bacterial diversity (**Supplementary Figure 2**). The microbiota of both ECRSwNP and NECRSwNP groups showed significantly decreased richness compared with the HC group (OTUs index and Chao1 index in **Figures 1A, B** **)**, however, the Shannon index significantly decreased only in the NECRSwNP group but not in the ECRSwNP group compared to the HC group (**Figure 1C**). The comparison between ECRSwNP and NECRSwNP groups did not reach a significant difference with regard to the observed OTUs, Chao1, and Shannon indexes. These results suggest that there are differences of alpha-diversity between HC and CRSwNP patients. Beta diversity by principal coordinate analysis **(**PCoA) summarizes compositional differences between, rather than within, microbial communities. ANOSIM analysis revealed significant differences in the community composition among the three groups. The further pairwise comparisons between groups revealed that while an overlap between ECRSwNP and NECRSwNP groups was observed, it was possible to underline statistically significant partitions between the HC group and both the ECRSwNP and NECRSwNP groups (HC *vs* ECRSwNP, *p*=0.0024; HC *vs* NECRSwNP, *p*=0.014) (**Figure 1D**).

### Sinus Microbiome Composition and Community Structure Alterations in Different CRSwNP Endotypes

**Figure 2:**
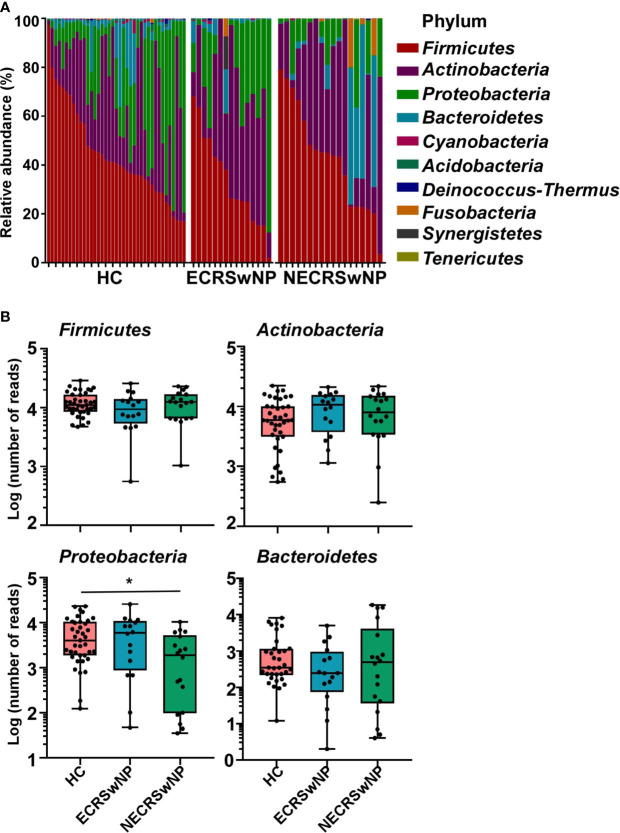
Relative abundance of major phyla in the sinus microbiome of single individuals and absolute abundance of major phyla among the groups. **(A)** Relative abundance of major phyla in the sinus microbiome of single individuals. **(B)** Absolute abundance of *Firmicutes*, *Actinobacteria*, *Proteobacteria*, and *Bacteroidetes* among the groups. Whisker boxes are drawn from the first to third quartiles. Error bars show minima and maxima. Statistical evaluations were performed using the Kruskal-Wallis test following by Dunn’s multiple comparisons test. ^*^ *p* < 0.05. HC, healthy control; ECRSwNP, eosinophilic chronic rhinosinusitis with nasal polyps; NECRSwNP, non-eosinophilic chronic rhinosinusitis with nasal polyps.

The main components of the sinus microbiota were first analyzed for the HC and CRSwNP groups. The sinus microbiome of all subjects was represented primarily by the phyla *Firmicutes, Actinobacteria, Proteobacteria, and Bacteroidetes* (**Supplementary Figure 3**). The proportion of *Firmicutes* was the highest in both the HC (44.65%) and CRSwNP groups (40.02%), *Actinobacteria* (26.49% in the HC group, 34.03% in the CRSwNP group) and *Proteobacteria* (23.16% in the HC group, 16.05% in the CRSwNP group) ranked, respectively, second and third. When CRSwNP patients were further divided into two endotypes, the top three phyla in the ECRSwNP group were *Firmicutes* (36.97%), *Actinobacteria* (35.11%), and *Proteobacteria* (23.51%). However, *Bacteroidetes* (12.51%) instead of *Proteobacteria* (9.39%), was the third most abundant phylum in the NECRSwNP group. We observed that the abundance of *Proteobacteria* was significantly lower in the NECRSwNP group compared to HC subjects **(** **Figure 2** **)**.

**Figure 3:**
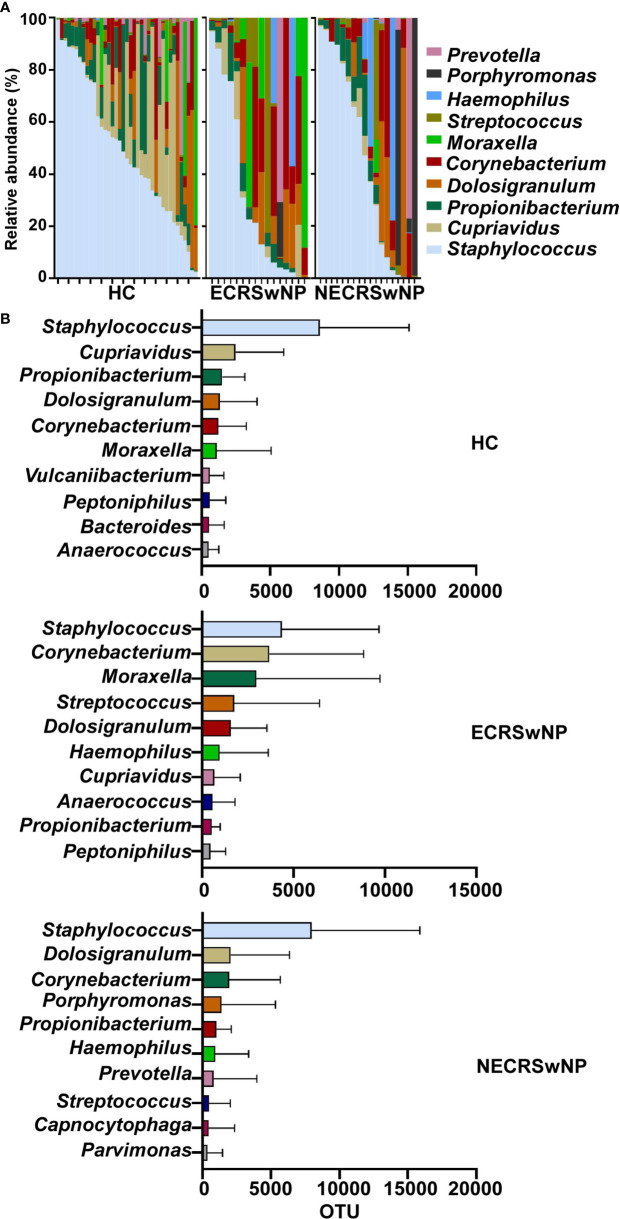
Relative abundance of major genera in the sinus microbiome of single individuals and absolute abundance of major genera among the groups. **(A)** Relative abundance of major genera in the sinus microbiome of single individuals. **(B)** Absolute abundance of major genera among the groups. Bars depict means; error bars, standard deviation (SD). HC, healthy control; ECRSwNP, eosinophilic chronic rhinosinusitis with nasal polyps; NECRSwNP, non-eosinophilic chronic rhinosinusitis with nasal polyps.

The characteristics and alterations in community structure of the sinus microbiota were further analyzed at the genus level. The magnitude and types of taxa in HC subjects and CRSwNP patients were considerably different (**Supplementary Figure 4**). The most abundant genera in healthy control subjects were *Staphylococcus*, followed by *Cupriavidus* and *Propionibacterium*. *Staphylococcus* dominated in CRSwNP patients, *Corynebacterium* and *Dolosigranulum* ranked second and third (**Supplementary Figure 4**). However, when the CRSwNP group was split into ECRSwNP and NECRSwNP, *Staphylococcus* was still the most abundant genera in the two endotypes. *Dolosigranulum* and *Corynebacterium* ranked second and third in the NECRSwNP group, whereas *Moraxella* was the top third genera in ECRSwNP patients (**Figure 3**). We should also pay attention to the potential pathogens including *Hemophilus* and *Streptococcus*, which were all listed as the top 10 genera in the CRSwNP but not in the HC group (**Supplementary Figure 4B**).

### Comparison of the Pathogenic Genus Abundances in Different CRSwNP Endotypes

**Figure 4:**
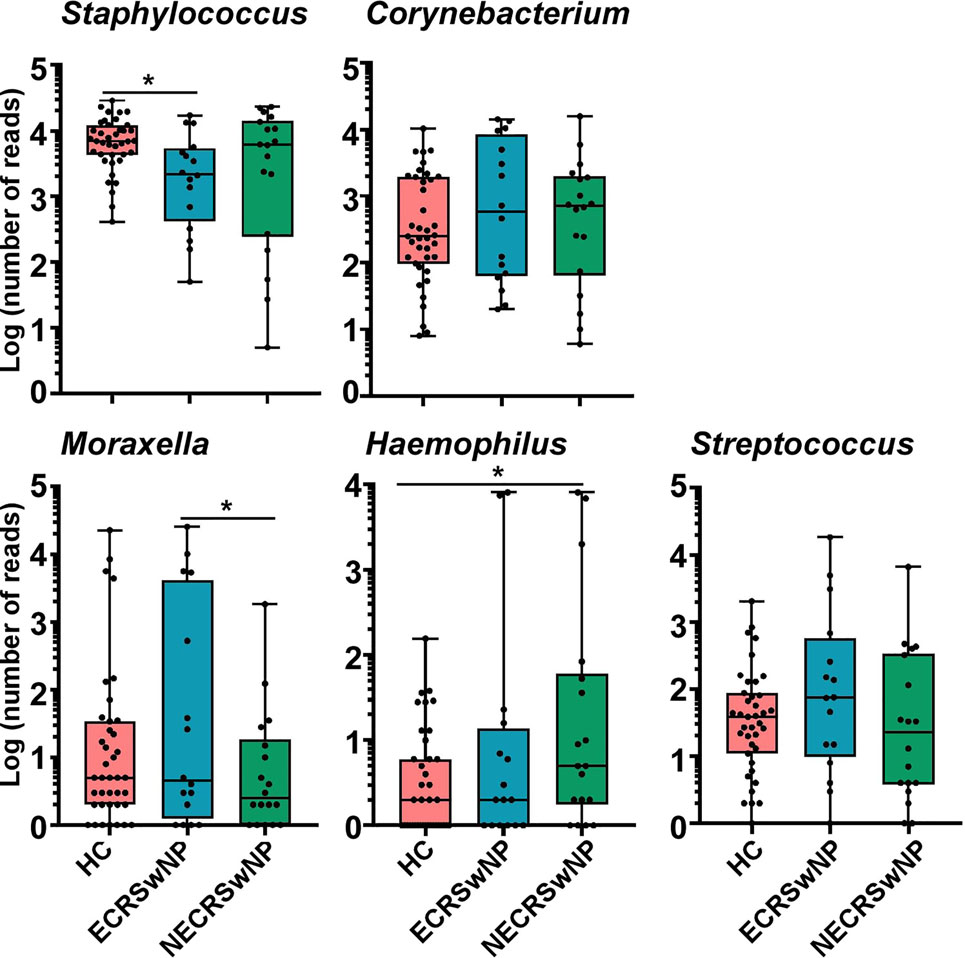
Absolute abundance of Staphylococcus, Corynebacterium, Moraxella, Hemophilus, and Streptococcus among groups. Whisker boxes are drawn from the first to third quartiles. Error bars show minima and maxima. Statistical evaluations were performed using the Kruskal-Wallis test following by Dunn’s multiple comparisons test. ^*^ *p* < 0.05. HC, healthy control; ECRSwNP, eosinophilic chronic rhinosinusitis with nasal polyps; NECRSwNP, non-eosinophilic chronic rhinosinusitis with nasal polyps.

Here, we focused on the most abundant genus and the prevalent pathogens including *Moraxella*, *Hemophilus*, and *Streptococcus.* We observed that the abundance of *Staphylococcus* was significantly lower in the ECRSwNP group compared to healthy control subjects (**Figure 4**). There were no significant differences in the abundance of *Corynebacterium* and *Streptococcus* among the three groups (**Figure 4**). The abundance of *Moraxella* was significantly higher in the ECRSwNP compared with the NECRSwNP group, while the abundance of *Hemophilus* was the most abundant in the NECRSwNP group (**Figure 4**).

### *S. aureus* Abundance Was Higher in NECRSwNP Patients

**Figure 5:**
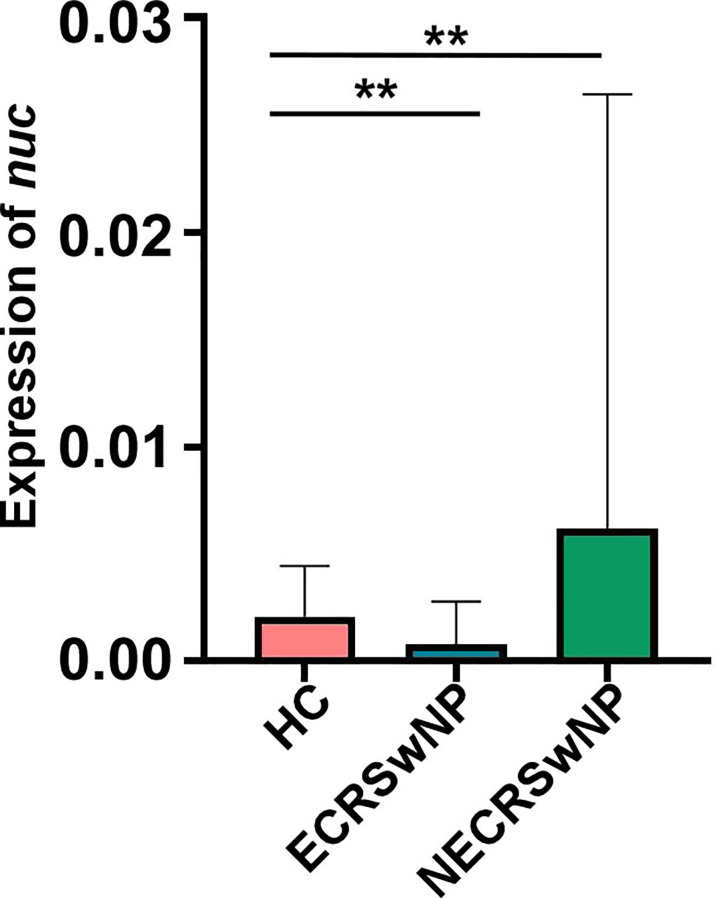
*S. aureus* burden among groups. Concentrations of *S. aureus* were determined using a TaqMan qPCR assay targeting the nuclease (*nuc*) gene. The results are presented as the mean ± SD. Statistical evaluations were performed using the Kruskal-Wallis test following by Dunn’s multiple comparisons test. ^**^ *p* < 0.01. HC, healthy control; ECRSwNP, eosinophilic chronic rhinosinusitis with nasal polyps; NECRSwNP, non-eosinophilic chronic rhinosinusitis with nasal polyps.

*Staphylococcus* is the top genus in both healthy adults and CRSwNP patients. *Staphylococci* include pathogenic *S. aureus* and coagulase negative staphylococci (CoNS). CoNS is generally beneficial and contributes to immune defense (Otto, 2004; Otto, 2009; Cogen et al., 2010; Grice and Segre, 2011). *S. aureus* infection and persistence is associated with CRS and it may be particularly relevant in CRSwNP. Several investigations found an increased colonization of *S. aureus* in CRSwNP patients (Van Zele et al., 2004; Vickery et al., 2019). Interestingly, we observed that the abundance of *Staphylococcus* was the lowest in the ECRSwNP group (**Figure 4**). To determine the exact composition of the *Staphylococcus* genus, the concentration of *S. aureus* was determined by Taqman qPCR targeting the nuclease (*nuc*) gene. Consistent with the results on a *Staphylococcus* genus level, we observed that the abundance of *S. aureus* was the lowest in the ECRSwNP group (**Figure 5**). Interestingly, although there was no significant difference of *S. aureus* abundance between ECRSwNP and NECRSwNP patients, which may attribute to the small sample sizes, the abundance of *S. aureus* was the highest in the NECRSwNP group, indicating that the colonization of *S. aureus* is involved in the persistent inflammation of NECRSwNP patients.

## Discussion

CRSwNP is a popular disease worldwide and seriously compromises quality of life. ECRSwNP and NECRSwNP are two major endotypes that are exhibit different clinical characteristics and have different therapeutic approaches. It has been reported that the microbiota is involved in the inflammation of CRSwNP (Hamilos, 2015; Hoggard et al., 2017b). However, the exact role of local microbiota in different endotypes is not identified. Because of the different infiltration of inflammatory cells, we hypothesized that variations in airway microbiome composition may exert distinct effects that contribute to CRSwNP heterogeneity. Here, we confirmed the significant differences of microbiome composition between CRSwNP patients and healthy adults. Importantly, we observed that the abundance of several important pathogenic bacteria including *Moraxella* and *S. aureus* are indeed significantly changed in different CRSwNP endotypes.

Lund-Mackay score and SNOT-22 are objective and subjective measurements of CRSwNP clinical manifestations. To quantify radiologic disease severity, patients with CRSwNP underwent sinus computed tomography scans and were evaluated by the Lund-Mackay score, with higher scores indicating a greater extent of disease. Our study shows that the ECRSwNP group had significantly higher Lund-Mackay scores of maxillary sinuses, anterior ethmoidal, posterior ethmoidal, frontal sinuses, and ostiomeatal complex compared with the NECRSwNP group, indicating that the ECRSwNP group presented with more severer clinical manifestations than the NECRSwNP group. However, the SNOT-22 test (high scores indicating worse symptoms), which was widely used for measurement of sinonasal symptomatology, did not display significant differences between ECRSwNP and NECRSwNP patients (**Table 2**). Previous studies demonstrated that ECRSwNP had significantly worse SNOT-22 scores than NECRSwNP (Thompson et al., 2016; Wang et al., 2019). The reason we did not observe the differences of SNOT-22 scores between the two groups is because only surgical CRSwNP patients with severe symptoms were included in the study. Further study should broaden the samples sizes with diverse severity.

Microbiome research has revealed that sinonasal microbiota play an important role in maintaining immune homeostasis. Bacterial community diversity was significantly lower in CRS samples compared to healthy subjects (Biswas et al., 2015). Moreover, bacterial diversity was found to be associated with postoperative outcomes (Ramakrishnan et al., 2015). This enhances the mucosal protective role and prevents the colonization of invading pathogenic bacteria. Thus, maintaining a rich diversity of sinus microbiota is highly beneficial in protecting against mucosal inflammation. Here, we also observed differences of community diversity between healthy adults and patients. For alpha-diversity index, both OTUs and Chao1 showed community richness, while the Shannon diversity took into account both community richness and evenness. We observed the OTUs, Chao1 and Shannon indexes were significantly higher in the HC group compared to the CRSwNP group (**Supplementary Figure 2** and **Figure 1**), indicating that the community diversity decreased in CRSwNP patients. The Shannon index in the HC group was significantly higher than that of the NECRSwNP group but not the ECRSwNP group (**Figure 1**), which suggested that the decreased diversity of sinus microbiota has greater impact in NECRSwNP patients. Moreover, we observed significant difference in beta-diversity (*p*=0.001, R=0.277) among different groups (**Figure 1**), indicating that the microbiota composition was diverse between CRSwNP patients and healthy adults.

*S. aureus* is a common colonizer of the human nose, skin, or gut. Approximately 20%-25% of the general population have persistent nasal colonization with *S. aureus* and 60% are an intermittent carrier (Bachert et al., 2002; Derycke et al., 2010; Krismer et al., 2017). However, the role of *S. aureus* in the pathogenesis of CRSwNP goes beyond mere colonization and is thought to play a specific role in the immunopathogenesis of CRSwNP. Several investigations found increased colonization of CRSwNP with *S. aureus*, which correlate with more severe disease phenotypes (Van Zele et al., 2004; Vickery et al., 2019). Chronic microbial infection or colonization in aged patients was associated with neutrophilic, rather than eosinophilic, tissue inflammation (Morse et al., 2019). NECRSwNP is associated with Th17 immune response, which is essential for protecting the host from various pathogens. The pathogenic *S. aureus* can induce Th17 response efficiently during infection (Cantero et al., 2015; Schirmer et al., 2016; Yu et al., 2021). Our data showed that the abundance of *S. aureus* in NECRSwNP patients is the highest among all groups (**Figure 5**), suggesting that the colonization of *S. aureus* may contribute to Th17 immune response during NECRSwNP. However, the abundance of *S. aureus* was determined by qPCR due to the limitation of 16S rRNA sequencing. Further study should implement the culture of all samples and confirm the role of *S. aureus* in the development of inflammation by animal tests.

*Moraxella* is a genera including many species. It is reported that the main proportion of *Moraxella* belonged to *M. catarrhalis* in asthma patients using whole metagenomic shotgun RNA sequencing (Castro-Nallar et al., 2015). In neonates, the colonization of the hypopharyngeal region with *M. catarrhalis*, member of genus *Moraxella*, has been reported as a potential risk factor for childhood asthma (Bisgaard et al., 2007). Importantly, *M. catarrhalis* contributes to diseases in the upper respiratory tract (Bisgaard et al., 2007). It is possible that *M. catarrhalis* is involved in CRSwNP. Here, we failed to identify these bacteria on a species level due to the limitation of 16S rRNA sequencing. However, the abundance of *Moraxella* was significant decreased in the NECRSwNP group compared with that in the ECRSwNP group. Although *M. catarrhalis* may contribute to chronic rhinosinusitis (Hoggard et al., 2017a; Bassiouni et al., 2020), *Moraxella* is not the main factor for NECRSwNP (**Figure 4**). Infants dominated by *Hemophilus* had higher rates of respiratory illness and a predisposition to asthma, likely due to the inflammatory potential of *Hemophilus* (Teo et al., 2015; Bosch et al., 2016). We observed that the abundance of *Hemophilus* was significantly increased in the NECRSwNP group compared to healthy control subjects (**Figure 4**), indicating that *Hemophilus* may play a role in the development of NECRSwNP (De Boeck et al., 2019). In conclusion, our study revealed that bacterial microbiome dysbiosis is present in both ECRSwNP and NECRSwNP patients in different patterns. Furthermore, the abundance of pathogenic bacteria is significantly different between ECRSwNP and NECRSwNP patients. Our results suggest that variations in airway microbiome structure and composition may exert distinct effects that contribute to CRSwNP heterogeneity.

Airway exposure to bacteria and their products has been shown to be potently effective in the suppression of allergic airway inflammation in adult mice (Nembrini et al., 2011; Hagner et al., 2013). A double-blind, placebo-controlled study investigated that probiotic supplementation effectively reduced fever and rhinorrhea and cough incidence against upper respiratory pathogens (Leyer et al., 2009). Maintaining a healthy microbiota provides a novel hypothesis for future studies on disease mechanisms. Limitations of this study include the fact that we did not do culture-based identification, which would have broadened a more comprehensive scale of sinus microbiota in healthy and inflammation states. Further investigations are needed to define the specific species of sinus bacteria involved in mucosa inflammation of CRSwNP.

## Data Availability Statement

The original contributions presented in the study are publicly available. This data can be found here: https://www.ncbi.nlm.nih.gov/, with accession number PRJNA697755.

## Ethics Statement

The studies involving human participants were reviewed and approved by the Research Ethics Committee of Renji Hospital. The patients/participants provided their written informed consent to participate in this study.

## Author Contributions

TF performed the experiments and wrote the manuscript. PM and BL analyzed the data. YaL, XB provided data analysis consultation. JX, NR and YiL collected samples. JS and WC provided critical review. JF performed the histological analysis. ML, QL and JP designed the study. All authors contributed to the article and approved the submitted version.

## Conflict of Interest

The authors declare that the research was conducted in the absence of any commercial or financial relationships that could be construed as a potential conflict of interest.

## Publisher’s Note

All claims expressed in this article are solely those of the authors and do not necessarily represent those of their affiliated organizations, or those of the publisher, the editors and the reviewers. Any product that may be evaluated in this article, or claim that may be made by its manufacturer, is not guaranteed or endorsed by the publisher.
