# Supplementary material for: Sinus Microbiota in Patients With Eosinophilic and Non-Eosinophilic Chronic Rhinosinusitis With Nasal Polyps
Source: Front Cell Infect Microbiol. 2021 Jul 23;11:672355. doi: 10.3389/fcimb.2021.672355 (PMC8346020; doi:10.3389/fcimb.2021.672355)
Supplement: Supplementary file 1 [file DataSheet_1.docx]

Supplementary Material


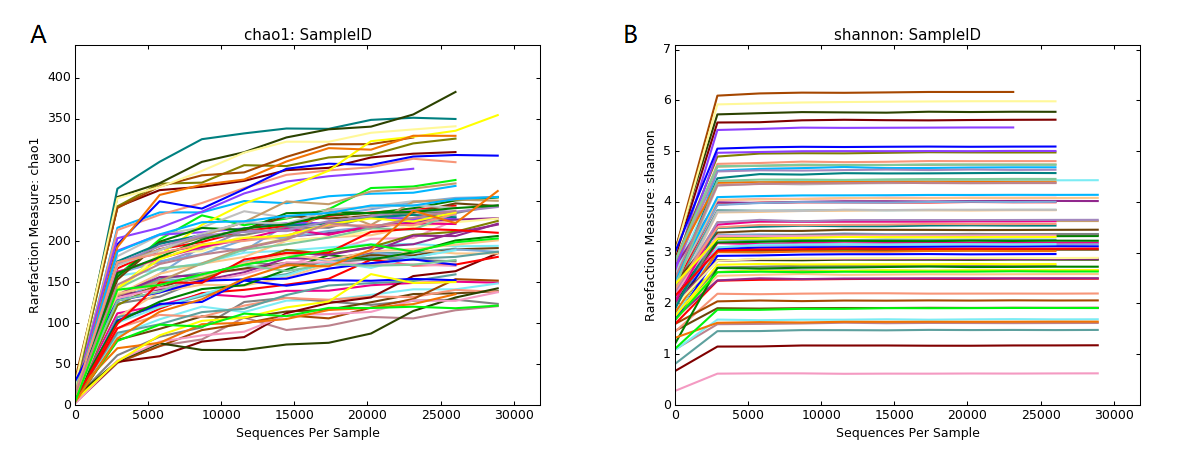


**Supplementary Figure 1.**The rarefaction curves of OTUs. (**A**) The rarefaction curves of chao1 index; (**B**) The rarefaction curves of shannon index. The x-axis shows the number of valid sequences per sample and the y-axis shows the observed OTUs. Each curve in the graph represents a different sample and is shown in a different color.

**
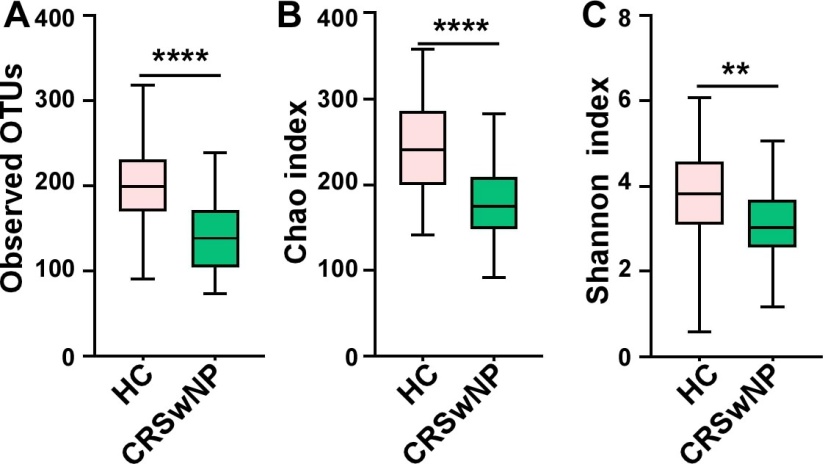
**

**Supplementary Figure 2. Differences in alpha diversity of the sinus microbiota between HC and CRSwNP groups. (A)** Number of observed OTUs;**(B)** Chao1 diversity index; **(C)** Shannon diversity index. n=39 for Healthy control, n=34 for CRSwNP. Whisker boxes are drawn from the first to third quartiles. Error bars show minima and maxima. ^**^*p*<0.01, ^****^*p*<0.0001, statistical evaluation is by unpaired *t* test. Abbreviations: HC: healthy control; CRSwNP: chronic rhinosinusitis with nasal polyps.


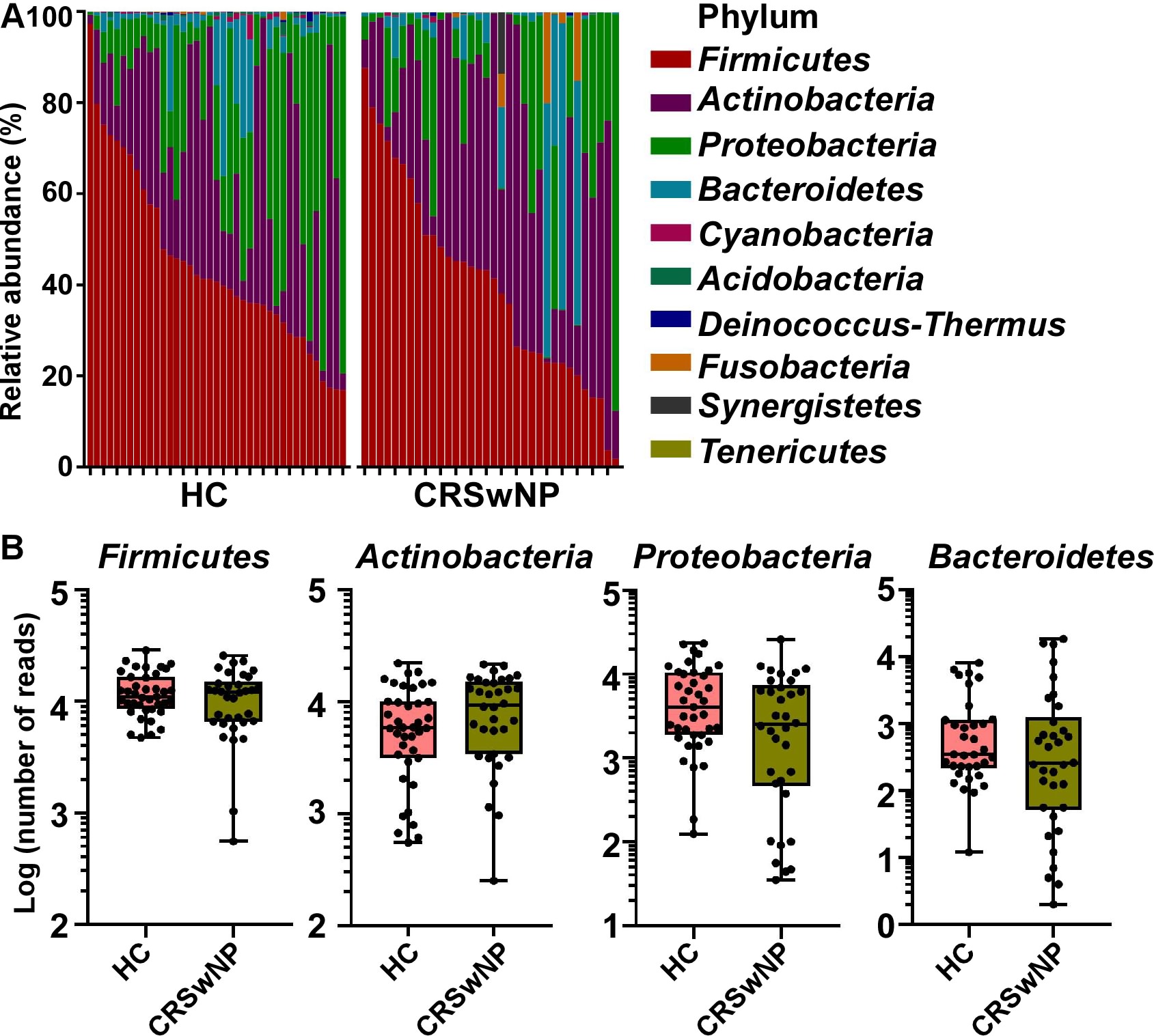
**Supplementary Figure 3. Relative abundance of major phyla in the sinus microbiome of single individuals and difference in absolute abundance of major phyla between HC and CRSwNP group. (A)** Relative abundance of major phyla in the sinus microbiome of single individuals. **(B)** Difference in absolute abundance of *Firmicutes*, *Actinobacteria*, *Proteobacteria*, *Bacteroidetes* between HC and CRSwNP group. Whisker boxes are drawn from the first to third quartiles. Error bars show minima and maxima. Statistical evaluations were performed using the Mann-Whitney test. Abbreviations: HC: healthy control; CRSwNP: chronic rhinosinusitis with nasal polyps.


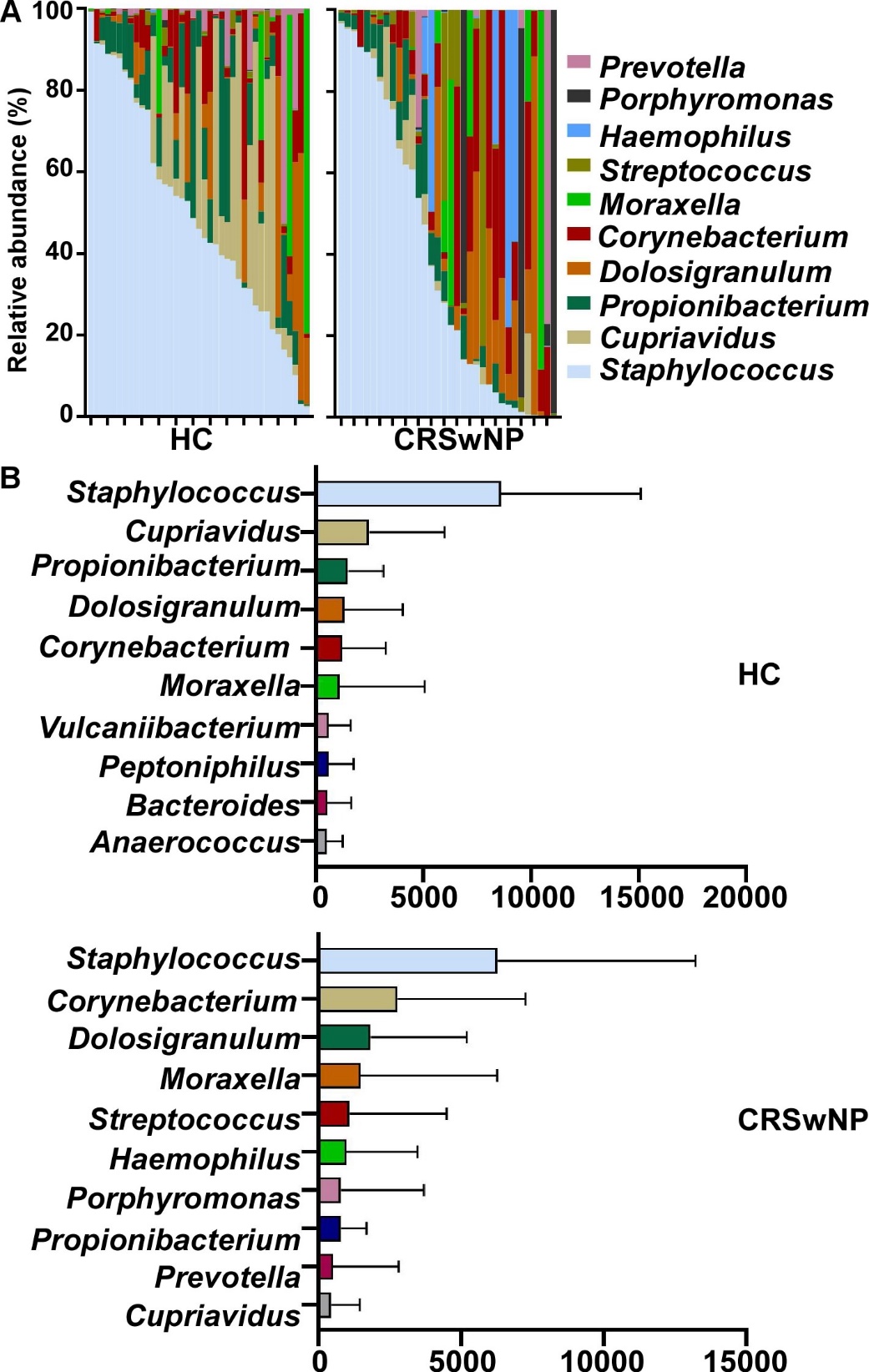


**Supplementary Figure 4. Relative abundance of major genera in the sinus microbiome of single individuals and absolute abundance of major genera in HC and CRSwNP group. (A)** Relative abundance of major genera in the sinus microbiome of single individuals. **(B)** Absolute abundance of major genera in HC and CRSwNP group. Bars depict means; error bars, standard deviation (SD). Abbreviations: HC: healthy control; CRSwNP: chronic rhinosinusitis with nasal polyps.

**Supplementary Table 1. Cytokine profiling of patients with ECRSwNP and NECRSwNP**

| Parameter | ECRSwNP | NECRSwNP | *p*-value |
| --- | --- | --- | --- |
| INF-γ | 1.23±0.76 | 1.43±0.67 | *p*=0.483 |
| TNF-α | 2.11±1.97 | 1.28±0.62 | *p*=0.582 |
| IL-2 | 1.36±0.78 | 1.06±0.39 | *p*=0.434 |
| IL-4 | 0.98±0.90 | 0.87±0.66 | *p*=0.735 |
| IL-6 | 3.12±2.11 | 2.28±1.08 | *p*=0.284 |
| IL-10 | 2.08±0.59 | 1.79±0.70 | *p*=0.290 |
| IL-17A | 4.15±2.70 | 3.27±4.63 | *p*=0.068 |

Data are the mean ± standard deviation (SD). Abbreviations: ECRSwNP: eosinophilic chronic rhinosinusitis with nasal polyps; NECRSwNP: non-eosinophilic chronic rhinosinusitis with nasal polyps; Statistically significant differences between groups were calculated using unpaired *t*-test or Mann-Whitney test. *p*< 0.05 is considered significant.
